# Which Species Should We Focus On? Umbrella Species Assessment in Southwest China

**DOI:** 10.3390/biology8020042

**Published:** 2019-05-23

**Authors:** Xuewei Shi, Cheng Gong, Lu Zhang, Jian Hu, Zhiyun Ouyang, Yi Xiao

**Affiliations:** 1 State Key Laboratory of Urban and Regional Ecology, Research Center for Eco-environmental Science, Chinese Academy of Sciences, Beijing 100085, China; xwshi_st@rcees.ac.cn (X.S.); luzhang@rcees.ac.cn (L.Z.); zyouyang@rcees.ac.cn (Z.O.); 2 University of Chinese Academy of Sciences, Beijing 100049, China; 3 School of Life Sciences, University of Science and Technology of China, Hefei 230026, China; cgong@mail.ustc.edu.cn; 4 Institute of Qinghai-Tibetan Plateau, Southwest Minzu University, Chengdu 610041, China; jianhu@swun.edu.cn

**Keywords:** umbrella species, biodiversity conservation, Southwest China, indicator value analysis, correlation analysis, factor analysis

## Abstract

In conservation biology, umbrella species are often used as agents for a broader set of species, or as representatives of an ecosystem, and their conservation is expected to benefit a large number of naturally co-occurring species. Southwest China is home to not only global biodiversity hotspots, but also rapid economic and population growth and extensive changes in land use. However, because of the large regional span, the diverse species distributions, and the difficulty of field investigations, traditional methods used to assess umbrella species are not suitable for implementation in Southwest China. In the current study, we assessed 810 key protected species from seven taxa by indicator value analysis, correlation analysis, and factor analysis. We selected 32 species as umbrella species, whose habitats overlapped the habitats of 97% of the total species. Furthermore, the selected species were significantly correlated with 70% of all species in the study area. A total of 16 out of 19 selected animal species have been previously mentioned as umbrella species, compared with only 3 out of 13 plants species; this is despite plants accounting for a large proportion of the total species in Southwest China. We discuss the roles of indicator species and co-occurring species, and provide suggestions for species protection in Southwest China based on the current results. Our research provides valuable scientific information for research on umbrella conservation species over large geographical scales, and related fields of biodiversity conservation.

## 1. Introduction

Species are not only the basic components of biodiversity, but also the basic units for biodiversity evolution, ecosystem function, and natural resource utilization [1,2]. Population trends, extinction risk, habitats, and condition of species, as well as community composition, can be used as indicators of biodiversity [3]. Thus, it is unsurprising that species have an important role in biodiversity conservation strategies. Currently, 25% of all mammalian species and 13% of all bird species, as well as more than 21,000 other species of plants and other animals, are threatened by extinction [4]. Current rates of extinction are ~1000 times the background rate of extinction, and are higher than those previously estimated [5].

In some regions, the number of species of concern is too large, biodiversity surveys are incomplete and it is difficult to consider each species on its own. In such situations, surrogate species or groups of species are often used as agents for a broader set of species, or as a representative of the state of a community or ecosystem to maintain and enhance biodiversity [6,7]. Faced with limited resources and increasingly larger challenges, conservation scientists have developed the umbrella species concept, whereby the conservation of one or more species is expected to confer protection to a large number of naturally co-occurring species [8,9], making conservation planning more attainable [10]. In addition to representing different aspects of biodiversity, the identification of a single umbrella species is limited by factors such as stochasticity, demography, phenology, and sampling efforts. Thus, habitat patches supporting high biodiversity can remain unoccupied by a single umbrella species [11]. However, rigorous assessments using multiple species show that umbrella species can be an effective tool for prioritizing remnants to be included in conservation networks [8].

Since 2000, China has made some progress in protecting and restoring its ecological assets, resulting in improvements in ecosystem services from 2000 to 2010; however, less progress has been seen in terms of biodiversity protection [12]. Given the spatial mismatch between the locations of conservation reserves and the distributions of threatened species [13], biodiversity conservation is difficult in China. However, the latest International Union for Conservation of Nature Red List (IUCN Red List) downgraded the status of some threatened species, such as the giant panda *Ailuropoda melanoleuca* and the Tibetan antelope *Pantholops hodgsoni* [14], suggesting that conservation efforts aimed at these species have at least been successful to a certain degree. The protection afforded to these species not only has positive effects on co-occurring species [8,15,16], but is also valuable for ecological restoration, and landscape planning and management [7,17,18]. However, in addition to these well-protected species, what other species should conservation efforts be focused on?

Southwest China is home not only to an important ecological dispersal barrier (the eastern Qinghai–Tibet Plateau) [19], but also a global biodiversity hotspot and a center for species differentiation [20,21], acting as a source of many ancient relict species. Over the past 20 years, Southwest China has experienced not only rapid socioeconomic development and extensive changes in land use, but also a series of problems, such as increasing threats to biodiversity and reductions in rare biological resources [4,22]. The competition between economic development and ecological environment protection is increasing in Southwest China and, thus, there is an urgent need for research on umbrella species in this region. Southwest China is considered as a whole in the Chinese administrative division and a key area in the protection of its biodiversity. Taking all of Southwest China as the study area is helpful to optimize arrangement for ecological protection and ecological construction and regional industrial development, to achieve coordinated development of the population, economy, resources, and environment. However, Southwest China covers a large area with a complex topography, changeable climate types, and large numbers of species with obvious differences in spatial distribution. Therefore, it is difficult to carry out large-scale field investigations to obtain detailed biological data of each species. This results in a significant challenge to research involving umbrella species in Southwest China.

In early studies, widespread species (mostly mammals and birds) tended to be selected as umbrella species [8]. When the study area and scale are small, this choice not only covers the habitats of co-occurring species, but also clearly reflects dynamic changes in the ecosystem [23]. However, species might be unevenly distributed over a large scale. Among them, widespread species, which have the ability to adapt to different habitats, appear in both biodiversity hotspots with high species richness and biodiversity coldspots, where only a few species occur, and can fail to clearly and accurately respond to local changes; in addition, there is a lack of conservation strategies that focus on protecting such widespread species [5]. Therefore, when the study area is large, it is necessary not only to select widespread species to measure changes in natural habitats, but also to identify species with aggregated distributions in local areas to observe its dynamics, especially in areas with high species richness.

At present, research on biodiversity conservation in China mainly focuses on the relationship between species hotspots and protected areas, as well as ecosystem services [13], the benefits of flagship species protection and their impacts on ecological function [15,24], and the impacts of land-use and/or cover changes on biodiversity [25]. Until now, there has been a lack of comprehensive assessments of umbrella species in China. Global studies have shown that the protection of umbrella species can not only have good diffusion benefits to their symbiotic species, but also be the core index of ecological restoration [17]. Research on umbrella species can reveal the necessary protection objectives, which can then be used to formulate a viable protection plan. Finally, the assessment of umbrella species can also enable scarce resources to be allocated in a more informed manner.

In this study, we collected data on 810 species mainly distributed in Southwest China. Based on the species spatial distribution characteristics, we selected our study area according to biodiversity indices and used indicator value analysis (IndVal analysis), correlation analysis, and factor analysis to select umbrella species based on three species characteristics (indicator values, representativeness, and spatial heterogeneity), combined with the level of importance of species protection. Finally, we discuss the characteristics of umbrella species, and provide suggestions for species protection strategies in Southwest China based on those characteristics.

## 2. Materials and Methods

### 2.1. Study Area

**Figure 1:**
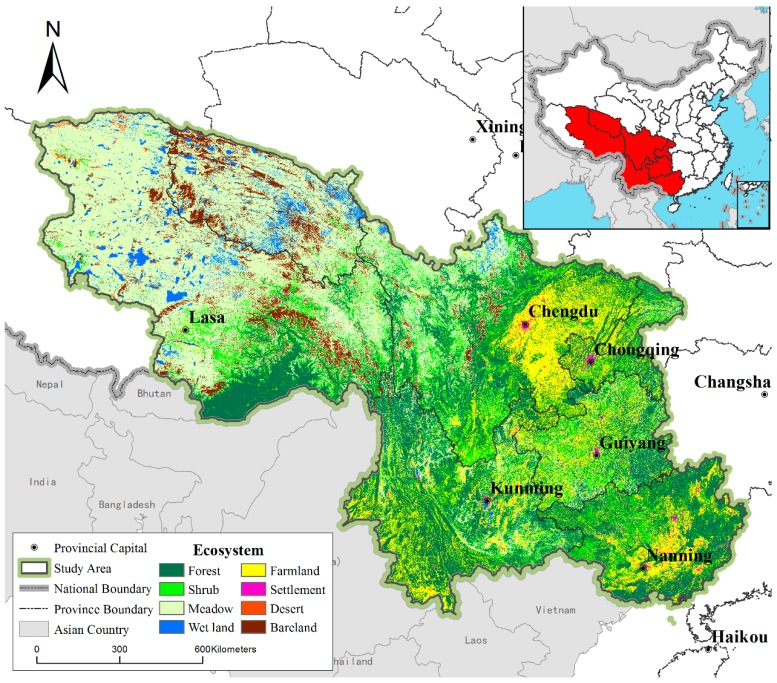
Study area.

The study area (Figure 1) included Guangxi, Chongqing, Sichuan, Guizhou, Yunnan, Southeast Tibet, and Southwest Qinghai (83°53′E–112°04′E,20°54′N–36°21′N), with a total area of 242.9 × 104 km^2^, encompassing 25% of the land surface of China. The types of climate include tropical rain forest, tropical subtropical monsoon, subtropical humid monsoon, subtropical evergreen broad-leaved forest, and plateau mountain climates. The annual temperature distribution is uneven but with obvious differences between the north and south of the region. The topography of Southwest China is complex and it includes the eastern part of the Qinghai–Tibet Plateau, the Yungui Plateau, Sichuan Basin, and Guangxi Basin. Landforms include plateaus, hills and karst landforms. The main types of ecosystems in the study area are forests, shrubs, and meadows, accounting for 73.6% of the total area.

### 2.2. Species List and Distribution

We selected species according to their listings in (i) The Checklist of National Protected Animals of China (2010) and The Checklist of National Protected Plants of China (2010) [26], including Class I and Class II (NPS); and (ii) threatened species (critically endangered, endangered, vulnerable) defined by the most recent IUCN Red List (2017) [14]. We sorted the species synonyms and subspecies according to the available data, and finally selected 810 species: 25 species of amphibians, 14 species of reptiles, 176 species of birds, 72 species of mammals, 23 species of bryophytes and ferns, 55 species of gymnosperms, and 445 species of angiosperms. Due to a lack of sufficient knowledge and data, the species in this study did not include invertebrates (Table A1).

Given that these species are threatened, endemic to China, and protected by law, they have high protection priority and represent the key protected species in Southwest China. We have rearranged them into three levels based on their conservation status [27] (Table 1).

Given the level of protection afforded to some species, only a few flagship species have precise spatial distribution data available, since most species are fuzzy and uncertain. We estimated the geographical range of each species based on information in the open databases and then modified that range based on ecological factors such as altitude and habitat, resulting in a potential habitat range for each species, a method that has already been used [13,15]. Species distribution, ecological requirements on elevation, and vegetation for each species were from Flora of China, the IUCN Red List, BirdLife International, Colored Atlas of Chinese Amphibians and Their Distributions, China’s Mammal Diversity and Geographic Distribution, The Reptile Database, and so on. We extracted elevational data using the 90-m digital elevation model from the NASA Shuttle Radar Topographic Mission, and vegetation data from the 30-m ecosystems map in 2010 [12,13]. By superposing the potential habitat of all species onto a map, we identified the spatial distribution pattern of key protected species.

We generated 1 km × 1 km squares as sample plots for this study. Using the tabulate area tool from Arcgis10.3, we calculated the potential habitat area of each species in the sample, resulting in a plot-by-species table. Based on this plot-by-species table, we calculated the presence and/or absence of each species. If the potential habitat area of a species in a plot was <5% of the total potential habitat, we considered this sample to reflect a marginal habitat for that species and the species was marked as absent from that plot, and vice versa. We calculated the species sample percentage (SSP) as the percentage of plots containing a species.

### 2.3. Statistical Analysis

#### 2.3.1. Indicator Species Analysis

The habitats of most species in Southwest China occur in forests, shrubs, meadows, and wetlands, accounting for 78% of the total study area. Some species habitats are similar to the area of concern, and protecting the habitat of these species means protecting the natural habitat of the study area, and at the same time protecting the habitat of other species. We assessed the relationship between species distribution and study area by IndVal analysis, which examines the species most closely related to the target area based on the indicator value index [28]. The species selected by IndVal analysis, which were called indicator species, not only reflect the change in ecosystem and indicate the region [29], but also overlap with most species in the same region.

The indicator value index is defined as the product of specificity A (or positive predictive value) and sensitivity B (or fidelity). Specificity, A = P(G|S), is defined as the probability that the surveyed site belongs to the target site group G given that species S has been found [30]; when species occur only in sites belonging to the target site group, A reaches a maximum of 1 [28]. Sensitivity, B = P(S|G), is defined as the probability that the species will be found in newly surveyed sites of the same target site group [30]. B reaches a maximum of 1 when a species is present in all sites of the target site group [28].

$$\sqrt{IndVal_{pa}}=\sqrt{A_{pa}\times B_{pa}}=\sqrt{\frac{n_{p}}{n}\times \frac{n_{p}}{N_{p}}}$$

N_p_, number of sites belonging to the target site group;

n, number of occurrences of the species among all sites;

n_p_, number of occurrences of the species within the target site group.

**Figure A1:**
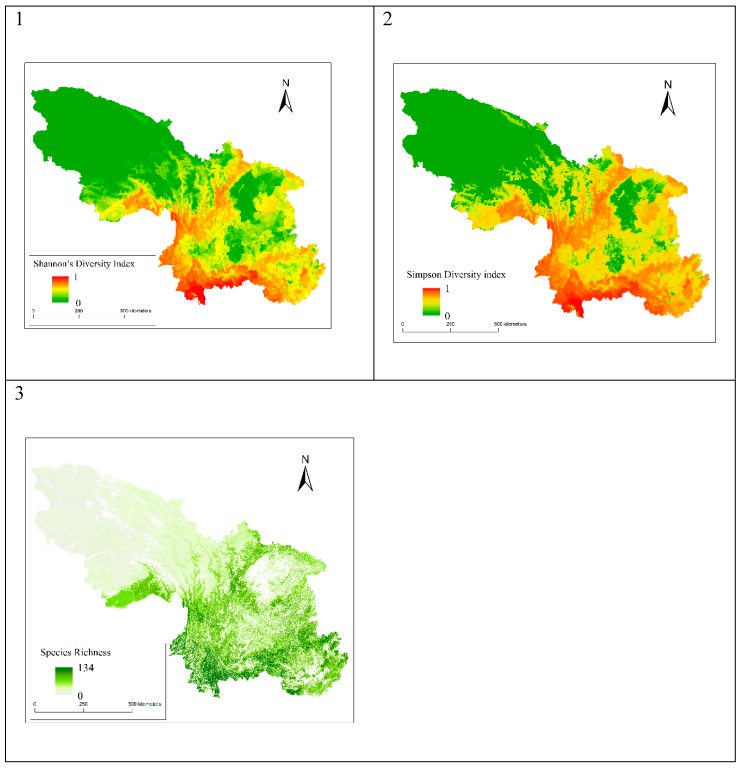
Biodiversity indices. (**1**) Shannon’s diversity index, (**2**) Simpson’s diversity index; (**3**) species richness.

IndVal analysis requires two inputs: (i) a plot-by-species table containing the presence–absence data; and (ii) partitioning of the plots into a set of plot groups (non-overlapping classes) [31]. Southwest China covers a large geographical area, and species richness in tropical regions is known to be significantly higher than that in other regions; therefore, the traditional partition method is not applicable to this region. On the basis of biodiversity indices (i.e., species richness, Simpson’s diversity index, and Shannon’s diversity Index, Figure A1), the study area was divided with reference to Chinese natural geographical regionalization and global ecological regionalization [32,33].

#### 2.3.2. Co-Occurring Species Analysis

The distribution of species in Southwest China is uneven, and includes not only hotspots of biodiversity, such as the mountainous areas of Southwest China, the Indo-Myanmar region, and Himalayan region, but also agriculturally developed areas, where species richness is low, such as the Sichuan Basin. As a result, additional attention should be paid to the area where species display aggregated distribution.

We used correlation analysis to characterize species aggregation. Two species are related when both are highly likely to be found in one sample plot or nonexistent at the same time. If a species has a large number of related species, it means that the species distribution is similar to many other species (i.e., they are co-occurring species). Consequently, the area where these species show aggregated distribution has great conservation value, and protecting the habitat of one species protects the habitat of others. To avoid selecting species distributing in the same high species-rich area, the spatial distribution of species was classified by factor analysis. Factor analysis groups several species with similar distribution into the same category. Species from a different category could represent species from different areas. Correlation analysis and factor analysis are used to determine the relationship between species, with the selected species being appropriate substitutions for co-occurring species.

##### Correlation Analysis

We used the Pearson’s square test to measure the correlation between two species and calculated the point correlation coefficient (PCC) to characterize the correlation between species. Given the large number of species combinations (810 × 810), only the correlation between *p* < 0.01 and a PCC >0.3 were considered (i.e., being extremely significant). We carried out chi-squared tests for each species and calculated the PCC to determine the correlation matrix. Finally, we tested the PCCs with Kruskal–Wallis and Mann–Whitney U Tests.

##### Factor Analysis

We used factor analysis to analyze the species spatial heterogeneity, and species were divided into groups based on their spatial distribution. Orthogonal rotation was chosen to enhance the interpretability of common factors, and factor scores were used to determine the degree of closeness of species codistributions. Factor analysis produced a number of different communities, and we only considered those communities in which the number of species in the taxa was greater than or equal to five.

### 2.4. Umbrella Species Selection

Indicator species were screened according to the indicator values. In each partition, we selected the species with a higher indicator value as the candidate species for further analysis if more than one species was chosen. Co-occurring species were selected according to the species correlation number and spatial distribution. The largest number of related species were selected in different groups as candidate species. Finally, the results of the two analyses were summarized and the umbrella species were screened if they met the criteria for selection.

## 3. Results

### 3.1. Biodiversity Spatial Distribution and Plot-by-Species Table

We obtained a spatial distribution of biodiversity in Southwest China by overlaying the potential habitats of 810 important protected species in this region. Forest was the most common habitat of these species. The hotspots of species distribution were located mainly in the tropical mountain forest regions of southeastern Tibet, the Qionglai–Minshan Mountains, the Daliangshan Mountains in Sichuan, the Hengduan Mountains in Yunnan, and tropical rain forests in southern to southeast Yunnan, Shiwan Mountains, Yunkai Mountains and Dayao Mountains in Guangxi, Miaoling Mountains, Leigongshan Mountains in Guizhou, and in other areas.

**Figure 2:**
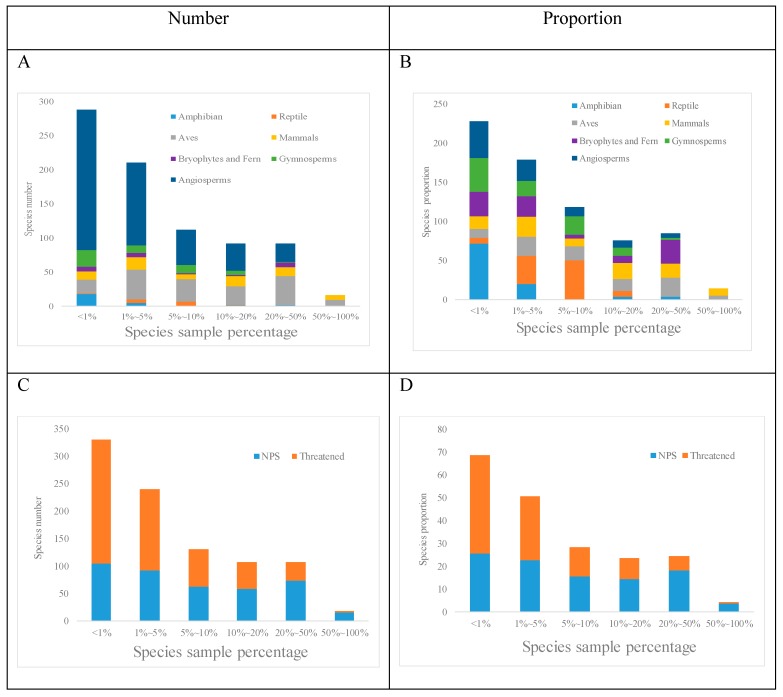
Species sample percentage for each taxonomic group. (**A**) Number of species in each taxon as a percentage of the species sample; (**B**) proportion of species in each taxon as a percentage of the species sample; (**C**) number of NPS and 0 species as a percentage of the species sample; (**D**) proportion for NPS and threatened species as a percentage of the species sample.

We calculated the species sample percentage (SSP) for each species. For 301 (37%) SSP <1%. This included 210 angiosperms, which were the most numerous of the taxa included in this study, and 72% of amphibians, which were the highest proportion of the taxa included in this study. There were 624 species with a SSP <10%, which accounted for 77% of the total species (Figure 2).

### 3.2. Statistical Analyses

#### 3.2.1. Indicator Species

**Figure 3:**
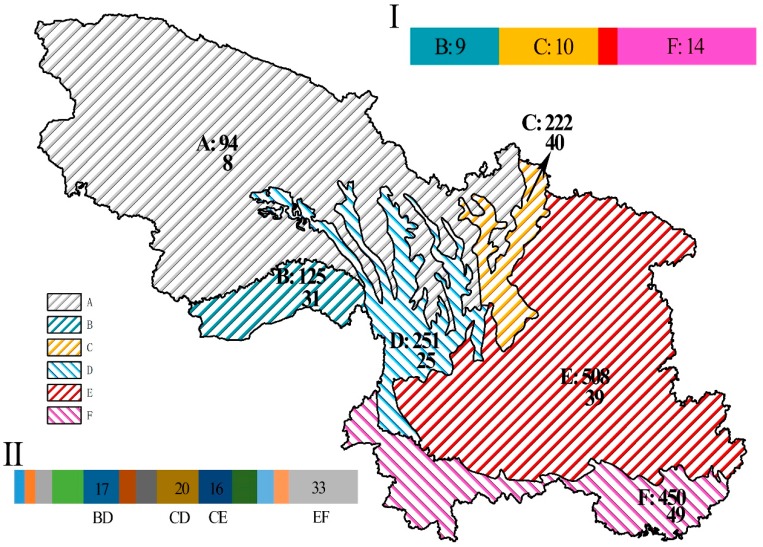
Division of research area. A: alpine shrublands and meadows; B: Eastern Himalayan broadleaf forests; C: Qionglai–Minshan conifer forests; D: Nujiang Langcang–Hengduan Mountains conifer and mixed forests; E: subtropical evergreen and mixed forests; F: tropical monsoon windy forest. Numbers next to each subregion refer to the number of species (top) and number of candidate species (bottom). (**I**) number of species that only indicate one subregion; (**II**) number of subregion combinations when species are indicative of multiple partitions.

On the basis of the biodiversity indices (i.e., species richness, Simpson’s diversity index, and Shannon’s diversity index, Figure A1), the study area was divided into six biodiversity regions: alpine shrub lands and meadows (A), Eastern Himalayan broadleaf forests (B), Qionglai–Minshan conifer forests (C), Nujiang Langcang–Hengduan Mountains conifer and mixed forests (D), subtropical evergreen and mixed forests (E), and tropical monsoon windy forest (F) (Figure 3). This partition yielded a total of 2^6^ − 1= 63 combinations for IndVal analysis. Only species with indicator values >0.6 were seen as candidate species, with 92 species finally being selected. The results showed that 35 species had a good indicative effect only in one region, mainly B (9), C (10), and F (14), implying that some species in these zones were isolated when compared with others. When the results pointed out more than one partition, such as partitions E and F, which often appeared together (33 times), indicating that some widespread species had no significant difference in terms of selecting these regions. There were 11 species with a good indicative effect on four or more subzones, most of which were not threatened but were still protected species (9). There were only eight species indicating region A, probably because: (1) fewer species occur in region A; and (ii) this region is very large.

We counted the number of species with habitats that overlapped with selected indicator species, and set up three scenarios to analyze the common habitats:

1: 40% habitat overlap: regarded as two species with a common habitat;
2: 60% habitat overlap: regarded as two species with a common habitat;
3: 80% habitat overlap: regarded as two species with a common habitat.

**Figure 4:**
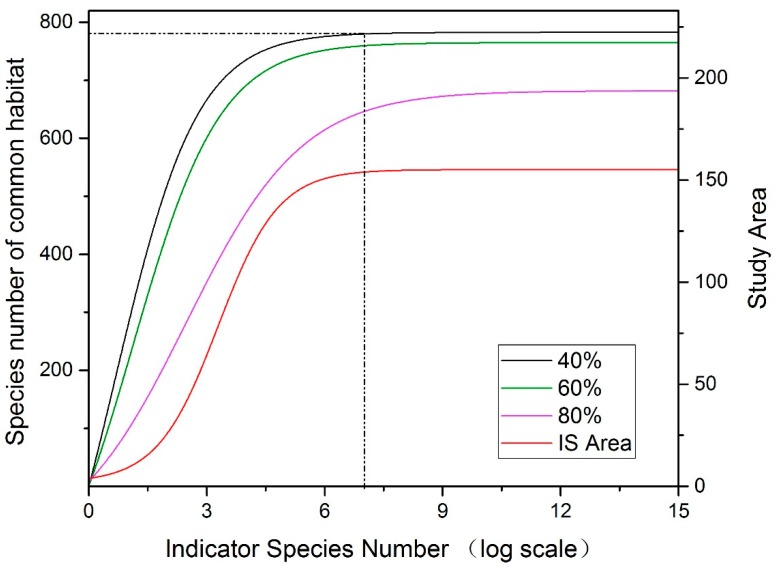
Number of species with habitats that overlapped with indicator species. IS area: indicator species habitat area.

As shown in Figure 4, 97% of species habitats were covered by seven indicator species under the 40% and 60% habitat overlap scenarios, with a habitat area of these indicator species of 153 × 104 km^2^, accounting for 65% of the study area. As the number of indicator species continued to increase, the curve remained unchanged under the 40% and 60% habitat overlap scenarios, but increased slightly under the 80% habitat overlap scenario.

Figure 4 also shows that the maximum number of indicator species to benefit was seven, although it is difficult for the theoretical model to reflect the different niche situations of different species in reality, and multi-categorized indicator species might have a more comprehensive indicative effect on the study area. Nevertheless, there is a direct relationship between the indicator value and the zoning method of the study area [34]. Therefore, the number of indicator species should increase appropriately according to the zonal characteristics and the indicating species category. According to the results of the IndVal analysis, 15 species were selected as candidate species, which were level 1 or level 2 protected species with good indicative function in different habitat zones. The candidate species list contained 8 species listed as both nationally protected species and threatened species (*Ailuropoda melanoleuca*, *Panthera uncia*, *Nycticebus bengalensis*, and so on), 3 species listed as nationally protected species but not threatened (*Aquila chrysaetos*, *Cymbidium aloifolium*, and *Sorolepidium glaciale*) and 4 species not listed in nationally protected species but threatened (*Eleutherococcus cuspidatus*, *Burretiodendron tonkinense*, *Aythya baeri*, and *Emberiza aureola*).

#### 3.2.2. Co-Occurring Species

**Figure 5:**
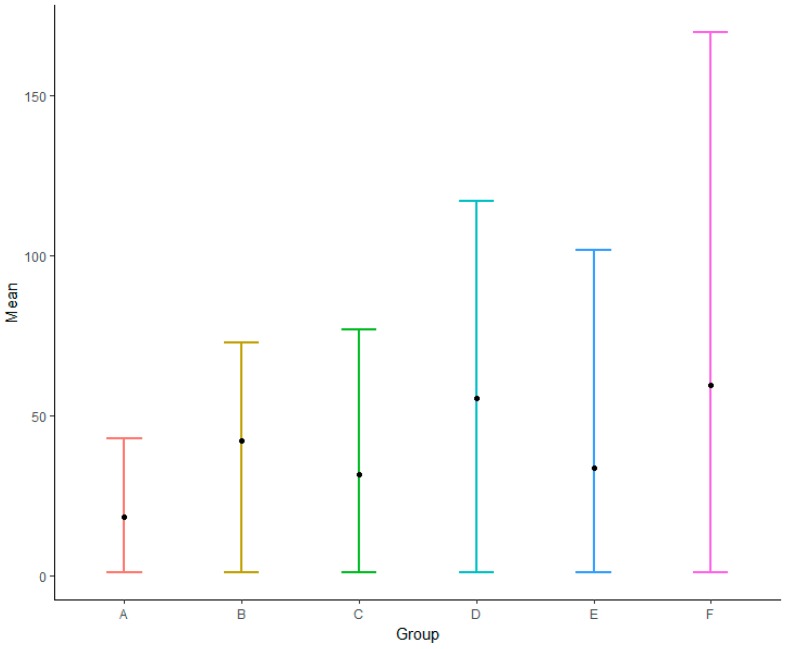
Mean effect size with 95% confidence intervals for the number of species with correlation relationships.

The correlation analysis of species was carried out in each zone (as detailed earlier), and the correlation matrix was arranged according to the number of related species. The number of species with correlation in region F was significantly higher than that in other subregions, and the number of species with correlation in region A was lower than that in other subzones (Figure 5). The most related species was the plant *Polyalthia verrucipes*, which was mainly distributed in southern Yunnan.

The number of related species directly reflects the species richness of the region. We analyzed the concentration of the distribution of the species area. Most of the related species in region A were concentrated mainly in the Ruoergai grassland and the conifer forest-grassland transition area. In region B, most of the related species were mainly concentrated in the Yarlung Zangbo Grand Canyon, whereas in region C, most related species were mainly concentrated in the south of the Qionglai Mountains and the Daliang Mountains. Most of the related species in region D were mainly concentrated in the Nu Mountains, Yunling Mountains, and Yulong Snow Mountains, whereas in region E, they were mainly concentrated to the southwest of the Wuling Mountains, Miaoling Mountains, western Nanling Mountains, and Dayao Mountains. Most of the related species in region F were mainly concentrated in the lower reaches of the Lancang River, Ailao Mountains, Liuzhao Mountains, and Shiwan Mountains.

The factor analysis produced a total number of 265 subgroups, with most subgroups (98) occurring in region E and the fewest (12) in region B. We selected the species with the highest number of related species from each group to represent that group. We selected 265 species that were used to analyze the final co-occurring species.

**Figure 6:**
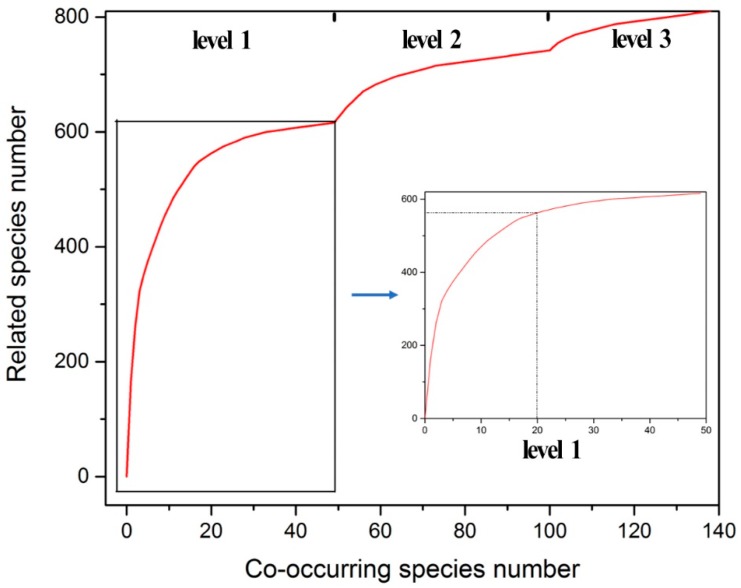
Correlation curve of the co-occurring species.

These species were ranked according to the protection level and the number of related species; the number of non-repetition-related species (i.e., the number of related species with all previous unduplicated species) was then ranked from high to low until all species in Southwest China were accounted for. In total, 138 species were counted, as shown in Figure 6. There were 49 species in level 1, which correlated with 76% of species and 100 species in levels 1 and 2, which correlated with 92% of species. When considering the number of species related to level 1 alone, 20 species were significantly correlated with 70% of species, indicating the greatest benefit of protecting these species. Therefore, we selected 20 co-occurring species as umbrella species. Sixteen of these species were listed as both nationally protected and threatened species (*Nyssa yunnanensis*, *Moschus berezovskii*, *Lophophorus lhuysii*, and so on) and four species were listed as nationally protected but not threatened species (*Nycticebus pygmaeus*, *Cycas balansae*, *Kingdonia uniflora*, and *Gypaetus barbatus*).

### 3.3. Umbrella Species Selection

Three species were selected from the combination of the two methods (indicator species analyses and co-occurring species analyses) detailed above; they were the giant panda *Ailuropoda melanoleuca*, red gazelle *Naemorhedus baileyi*, and bee monkey *Nycticebus bengalensis*, which gave umbrella protection to 32 species (Table 2 and Table A2).

The umbrella species mainly comprised of mammals, angiosperms, and birds, accounting for 34%, 25%, and 19% of the total species, respectively. Since mammals had a higher number of species, given that they have a wider range of habitat types than other species, they could better serve as regional umbrella species. In addition to common species, we included amphibians and reptiles. We chose reptiles (i.e., the Chinese three-striped box turtle (*Cuora trifasciata*)), since it was mainly distributed in mountainous streams of the Guangxi Province. This distribution pattern differs from other forest species, and the number of related species was also higher, including 50% of reptiles. Therefore, we chose it as co-occurring species. The amphibian, Chinese giant salamander (*Andrias davidianus*), was selected because it was mainly distributed in Sichuan, Chongqing, Yunnan, Guizhou, and Guangxi, and had a good indicative effect in region E.

## 4. Discussion

In the current study, we explored methods to assess umbrella species over a large geographical scale, adding to our understanding of umbrella species selection research in China. We used a combination analysis of indicator species and co-occurring species to evaluate umbrella species in Southwest China. Biodiversity indices were used to divide the study area, whereas IndVal analysis, correlation analysis, and factor analysis were used to describe the characteristics of species indications, correlations, and spatial heterogeneity. The umbrella species were chosen based on habitat overlap, non-repetition species correlation, and the level of species protection.

At region A only one indicator species (*Panthera uncia*) and two co-occurring species (*Lophophorus lhuysii* and *Gypaetus barbatus*) were selected. *Lophophorus lhuysii* is concentrated mainly in the Ruoergai grassland and the conifer forest-grassland transition area, where there is the highest species richness of region A. In region B, five species were selected as indicator species (*Panthera tigris*, *Budorcas taxicolor*, and so on), and one species was selected as both indicator species and co-occurring species (*Naemorhedus baileyi*), which were mainly concentrated in the Yarlung Zangbo Grand Canyon. Whereas in region C, nine species were selected as indicator species (*Eleutherococcus cuspidatus*, *Ailurus fulgens*, and so on), and one species was selected as both an indicator species and co-occurring species (*Ailuropoda melanoleuca*), and was mainly concentrated in the Minshan and the Qionglai mountains. In region D four indicator species (*Aquila chrysaetos*, *Ailurus fulgens*, and so on) and five co-occurring species (*Cycas bifida*, *Moschus berezovskii*, and so on) were selected. *Moschus berezovskii* was mainly concentrated in the Nu Mountains, Yunling Mountains, and Yulong Snow Mountains, where the highest species richness of region D was located. In region E, four species were selected as indicator species (*Andrias davidianus*, *Emberiza aureola*, and so on), and four species were selected as co-occurring species (*Rhinopithecus roxellana*, *Cuora trifasciata*, and so on). In region F, four species were selected as indicator species (*Burretiodendron tonkinense*, *Cymbidium aloifolium*, and so on), eight species were selected as co-occurring species (*Nyssa yunnanensis*, *Nycticebus pygmaeus*, and so on), and one species was selected as both indicator and co-occurring species (*Nycticebus bengalensis*) (Table A2).

Our results included 32 species, 19 of which either have been explicitly classified as umbrella species in previous studies, or the dynamics of which have a direct response to habitat changes or ecosystem management [15,16,35,36,37,38,39,40,41,42,43,44,45,46,47,48,49,50,51] (Table A2). Among these species, mammals had the highest proportion of umbrella species (10 out of 11, the only exception being the red gazelle *Naemorhedus baileyi*), followed by birds (four out of six birds). Although both amphibians and reptiles were represented only by one species on the list, they have also been shown to be indicative of habitat. The proportion of plants classified was low (two out of eight angiosperms, one out of three gymnosperms, and no bryophytes and ferns). Unsurprisingly, mammals and birds dominated as suggested, or evaluated, as umbrella species. However, according to the results of our study, plants accounted for a large proportion of the total species in Southwest China, with the most relevant species being Yunnan blue fruit trees (*Nyssa yunnanensi*). This suggests that it would be appropriate to select certain plants as umbrella species, although further studies are required to assess the impact of umbrella plant species on conservation.

We identified umbrella species in Southwest China based on indicator species versus co-occurring species. Indicator species were screened out based on the relationship between species distribution and the region, and the selected species were indicative of the region. Co-occurring species were selected according to the species correlation and spatial distribution characteristics, and the chosen species were representative of species in a community. Moreover, sensitivity (B) in IndVal analysis placed much weight on common species in each region, whereas the correlation analysis and factor analysis tended to select representative species in different communities with high species richness. Thus, indicator species tended to be found in each community in corresponding regions, whereas co-occurring species were the most representative species in different communities.

In general, indicator species and co-occurring species are often different species. However, when the study area contains a large number of indicator species and multiple communities, the two species may be the same. This is due to the higher correlation number of indicator species when there are more indicator species. In a community, if the correlation number of indicator species is greater than other species, it is very possible for the indicator species to be selected as the community representative species, which means that it is also a co-occurring species. For example, the red goral *Naemorhedus baileyi* in region B, giant panda *Ailuropoda melanoleuca* in region C, and bengal slow loris *Nycticebus bengalensisin* in region F are all indicator species and co-occurring species.

In situ conservation is one of the most effective measures in biodiversity conservation, but it is not suitable for each umbrella species. For co-occurring species, the area and quality of habitat, the connectivity between habitats, and the influence of human activities are the key factors for species protection [24]. For indicator species, the improvement of habitat quality and the management of existing conservation systems will bring more benefits. Thus, we recommend that co-occurring species should be selected as the main protection target, combining the habitat distribution of the species, corridor construction, ecosystem services, and human disturbances to establish strictly protected areas. Most indicator species have significant value in ecological restoration, landscape planning and so on; therefore, it is necessary not only to use these species as ecological evaluation indicators, but also to improve habitat quality and management of the existing network of protected areas.

According to the selection principle for umbrella species, such species tend to be either widely distributed across or concentrated within pockets in the study area. However, this approach neglects species that are distributed in particular regions or habitats. Our results showed that 27% of the species had no significant correlation with the identified umbrella species, although their habitats overlapped. This is because the habitat patterns of these species differ from those of umbrella species (too small or too large), and so it is difficult to reflect dynamic changes in these species. Thus, different conservation strategies should be considered for such species.

The method used to divide the study area may have had an impact on the final outcome. We used biodiversity indices to divide the study area based on Chinese natural geographical regionalization and global ecological regionalization, and to roughly classify different ecosystem types and different species richness regions. However, in practice, mountain systems, watersheds, and human disturbance also affect species distributions. When relevant data are available, future studies should consider a variety of factors to carefully select the most appropriate zoning method.

In this study, we only considered species in the first and second level of conservation importance when selecting indicator species because the range of indicator species habitats in the study area was unclear, and the habitat types were difficult to modify in detail because of the limitations of research methods. In addition, data associated with habitat distribution were poor. Similarly, third level species (i.e., vulnerable species or not threatened second protected species) received less research attention and, therefore, updates on such species were slow to emerge. In the current study, species presence and/or absence data were inferred from species habitat distributions, which can be simulated by models. This method is more appropriate to be used in study areas with a large number of species and a large geographical area. In addition, it does not have field investigations, reducing the length of research time required. Although species habitat distributions are closely related to ecosystem types and can directly reflect ecosystem dynamics, this method neglects various pieces of information, such as time series, season, phenology, and species behavior. Thus, it can overestimate the distributions of some species with wide habitat types, a large family range, or a small number of individuals. Given that it is difficult to reveal interspecific relationships between species based on non-field survey data used in statistical analysis, here we have only estimated the correlation between symbiotic species.

## 5. Conclusions

The value of the umbrella species concept for biodiversity conservation is well-known [10]. In recent years, an increasing amount of research has been done on the selection of umbrella species from multiple perspectives (e.g., ecological neighborhoods [9] and landscape connectivity [52]). However, controversy remains about how to select umbrella species [9,10,17,31,52,53,54], especially in geographically large areas. Authors have reported that rare species can also be used as umbrella species [54], but most rare species are difficult to observe, monitor, and collect data on. Therefore, we analyzed the presence and/or absence of species in samples based on simulated species habitat data. IndVal analysis, correlation analysis, and factor analysis were used to assess 810 important species (524 of which are in urgent need of protection) on a large scale across Southwest China to select umbrella species. Our research revealed that the relationship between umbrella species selection and division of the study area defined the key protection species and made suggestions for species protection in Southwest China. It also provided valuable scientific information for future research into the large-scale conservation of umbrella species and biodiversity in general.

## Figures and Tables

**Table 1:** Classification of species according to the level of their conservation status.

Evaluation Index	Conservation Status Classification Criteria		
	Level 1	Level 2	Level 3
Threatened	species that are both NPS and CR/EN	Other CR/EN species	VU species
Conservation level	Other first-category nationally protected species	---	Other second-category nationally protected species
Endemism	Narrow endemic species	Endemic to province species	Others species
Total	136	235	439

CR: critically endangered; EN: endangered; VU: vulnerable, defined by the most recent IUCN Red List; NPS: contains Class I and Class II category national protected species.

**Table 2:** Umbrella species composition.

Category	Amphibian	Reptile	Birds	Mammals	Bryophytes and Ferns	Gymnosperms	Angiosperms	Total
Indicator species	1	0	3	7	1	0	3	15
Co-occurring species	0	1	3	7	1	3	5	20
Total	1	1	6	11	2	3	8	32 ^1^
Proportion (%)	3	3	19	34	6	9	25	100

^1^ Three species were selected by the two methods at the same time.

## Appendix A

**Table A1:** Summary of key protected species.

No. of Important Species		Category Information			Total
		Endangered	Protected in Law	Both Endangered and Protected in Law	
Animals	Amphibian	21	6	2	25
	Reptile	12	7	5	14
	Aves	57	138	19	176
	Mammals	42	69	39	72
Summary of animals		132	220	65	287
Plants	Bryophytes and Fern	17	7	1	23
	Gymnosperms	44	37	26	55
	Angiosperm	331	143	29	445
Summary of plants		392	187	56	523
Total		524	407	121	810

**Table A2:** List of umbrella species in Southwest China.

Scientific Name	Category	Taxa	Region	Mentioned before	Endemic to China	IUCN Red List Category	NPS Category	Importance Level	Indicator Value Index	Non-Repetition Correlation
*Nyssa yunnanensis*	co-occurring species	Angiosperms	F	YES	√	CR	I	1	0.36	164
*Neocheiropteris palmatopedata*	co-occurring species	Bryophytes and Fern	D	NO	√	EN	II	1	0.48	98
*Carya sinensis*	co-occurring species	Angiosperms	F	NO	×	EN	II	1	0.55	60
*Magnolia grandis*	co-occurring species	Angiosperms	F	NO	√	CR	II	1	0.15	29
*Cycas bifida*	co-occurring species	Gymnosperms	D	NO	×	VU	I	1	0.22	24
*Moschus berezovskii*	co-occurring species	Mammals	D	YES	×	EN	I	1	0.58	20
*Nycticebus bengalensis*	co-occurring species/indicator species	Mammals	F	YES	×	VU	I	1	0.60	15
*Naemorhedus baileyi*	co-occurring species/indicator species	Mammals	B	NO	×	VU	I	1	0.63	20
*Lophophorus lhuysii*	co-occurring species	Aves	A	YES	√	VU	I	1	0.55	21
*Rhinopithecus roxellana*	co-occurring species	Mammals	E	YES	√	EN	I	1	0.54	18
*Bretschneidera sinensis*	co-occurring species	Angiosperms	E	YES	×	EN	I	1	0.58	16
*Cuora trifasciata*	co-occurring species	Reptile	E	YES	×	CR	II	1	0.42	12
*Nycticebus pygmaeus*	co-occurring species	Mammals	F	YES	×	N/A	I	1	0.15	11
*Ailuropoda melanoleuca*	co-occurring species/indicator species	Mammals	C	YES	√	VU	I	1	0.80	11
*Tragopan caboti*	co-occurring species	Aves	E	YES	√	VU	I	1	0.21	10
*Cycas debaoensis*	co-occurring species	Gymnosperms	F	YES	√	CR	I	1	0.18	11
*Cycas balansae*	co-occurring species	Gymnosperms	F	NO	×	Not Threatened	I	1	0.26	8
*Neofelis nebulosa*	co-occurring species	Mammals	D	YES	×	VU	I	1	0.45	5
*Kingdonia uniflora*	co-occurring species	Angiosperms	D	NO	√	Not Threatened	I	1	0.53	5
*Gypaetus barbatus*	co-occurring species	Aves	A	NO	×	Not Threatened	I	1	0.52	5
*Panthera tigris*	indicator species	Mammals	B	YES	×	EN	I	1	0.87	0
*Aquila chrysaetos*	indicator species	Aves	B\C\D	YES	×	Not Threatened	I	1	0.73	0
*Panthera uncia*	indicator species	Mammals	A\C\D	YES	×	EN	I	1	0.68	0
*Cymbidium aloifolium*	indicator species	Angiosperms	E\F	NO	×	Not Threatened	I	1	0.68	0
*Andrias davidianus*	indicator species	Amphibian	E	YES	√	CR	II	1	0.65	0
*Sorolepidium glaciale*	indicator species	Bryophytes and Fern	B\C\D	NO	√	Not Threatened	I	1	0.64	0
*Budorcas taxicolor*	indicator species	Mammals	B\C	YES	×	VU	I	1	0.64	0
*Ailurus fulgens*	indicator species	Mammals	B\C\D	YES	×	EN	II	1	0.62	0
*Eleutherococcus cuspidatus*	indicator species	Angiosperms	C	NO	×	EN	Not Listed	2	0.73	1
*Burretiodendron tonkinense*	indicator species	Angiosperms	F	NO	×	EN	Not Listed	2	0.70	0
*Aythya baeri*	indicator species	Aves	E\F	NO	×	CR	Not Listed	2	0.62	0
*Emberiza aureola*	indicator species	Aves	C\E\F	YES	×	EN	Not Listed	2	0.60	0
